# Physicochemical Properties of Curen® Filaments Versus Nylon Filaments in Toothbrush Bristles: An In Vitro Study

**DOI:** 10.7759/cureus.75767

**Published:** 2024-12-15

**Authors:** PSG Prakash, Jasmine Crena M, Kriti Kaushik, Kirti Shukla, Sunil Kumar Yadav Yadagiri, Kranthi Kiran Pebbili, Gauri Dhanaki, Bhavesh P Kotak

**Affiliations:** 1 Periodontology, SRM Dental College, Chennai, IND; 2 Medical Affairs, Dr. Reddy's Laboratories Ltd., Hyderabad, IND; 3 Clinical Research, Dr. Reddy's Laboratories Ltd., Hyderabad, IND; 4 Medical Affairs, Dr. Reddy’s Laboratories Ltd., Hyderabad, IND

**Keywords:** abrasion, dental plaque, oral hygiene, tensile strength, toothbrushing

## Abstract

Background

Toothbrush manufacturers commonly use bristle materials such as nylon, polybutylene terephthalate, polypropylene, polyethylene terephthalate, boar hair, bamboo, carbon fiber, silicone, polylactic acid, or their modifications such as Curen^®^. Nylon filaments have long been demonstrated to be durable and are widely used, but not much is known regarding the performance of Curen^®^ filaments compared to nylon filaments. This in vitro study compared the stiffness, abrasion potential, abrasion resistance, and bristle surface changes of Curen^®^ and nylon filaments.

Methodology

Ten specimens (five dry and five wet) each of Curaprox CS5460 toothbrushes featuring Curen^®^ filaments and those with nylon filaments were subjected to tensile strength and force-displacement analyses. Brushing simulation (1,000, 2,000, 3,000, and 5,000 cycles) was conducted using six freshly extracted central incisors (three specimens each for the Curen^®^ and nylon filament groups). Pre- and post-brushing simulation parameters included filament abrasion potential (atomic force microscopy of extracted tooth surface), filament abrasion resistance (field emission scanning electron microscopy), and bristle surface changes (stereomicroscopy and micro- and nano-computed tomography).

Results

Curen^®^ filaments exhibited lower tensile strengths (41.69 MPa [dry] and 35.18 MPa [wet]) than nylon filaments (321.56 MPa [dry] and 325.44 MPa [wet]), indicating that Curen^®^ filaments have lower abrasion potential (87% [dry] and 89% [wet]) and cause less mechanical wear of enamel, thereby resulting in a gentler cleaning experience compared to nylon filaments. Furthermore, the enamel surface roughness in the crown region decreased by 19.4% with the use of the Curen^®^ filaments, whereas it increased by 92.3% with the use of nylon filaments, indicating that Curen^®^ filaments are 72.84% less abrasive to enamel than nylon filaments. After 5,000 cycles of brushing simulation, Curen^®^ filaments showed 30% less splaying than nylon filaments, highlighting the longevity of Curen^®^ filaments up to six months of tooth brushing, which is twice the longevity of nylon filaments. There was a minimal decrease in height (12.0 mm to 11.95 mm, -0.4% change), an increase in top diameter (2.157 mm to 2.390 mm, 10.8% change), and a rise in base diameter (1.784 mm to 2.035 mm, 14% change) in the Curen^®^ filaments group. Taken together, these results indicate that Curen^®^ filaments are superior to nylon filaments as teeth-cleansing agents.

Conclusion

The findings of this in vitro analysis demonstrate the lower tensile strength and lesser abrasion potential of Curen^®^ filaments when compared with nylon filaments. Thus, Curen^® ^filaments cause fewer microscratches and abrasion of enamel when compared with nylon filaments, occurring due to day-to-day mechanical wear because of improper brushing technique. Furthermore, the lower tensile strength of Curen^®^ filaments provides greater flexibility, facilitating more effective cleaning of hard-to-reach areas compared to nylon filaments. Additionally, the lesser splaying of Curen^®^ filaments highlights their longevity, demonstrating that Curen^®^ filaments last twice as long as nylon filaments under regular brushing conditions. Based on these advantages, toothbrushes with Curen^®^ filaments should be a preferred choice over nylon filaments.

## Introduction

According to the World Health Organization (WHO), globally there were 1.09 billion prevalent cases of chronic periodontal disease and 91.5 million incident cases in 2019 [1]. The estimated cases of dental caries and periodontitis in the Indian population as per the WHO’s Global Oral Health Status Report 2019 were 0.36 billion and 0.22 billion, respectively [2]. Effective dental plaque control is crucial in maintaining periodontal health and preventing caries [3]. Tooth brushing is an effective mechanical method that serves as a primary and cost-effective tool for controlling plaque-associated diseases or tooth decay when performed following an effective technique and for optimal duration [4,5]. However, along with proper technique, toothbrush design also plays a crucial role.

Numerous toothbrush designs and supplementary devices have been developed [5]. An ideal toothbrush should have a flexible neck, acceptable bristle shape (free of sharp or jagged edges and endpoints), durable material (bristles and handle), and accessibility to all surfaces of teeth [6]. Studies have revealed that toothbrush bristle modifications affect plaque index. A bi-level toothbrush with feathered bristles enhances subgingival access by 35.7% and improves cleaning effectiveness by 54.5% compared with an identical bi-level brush with rounded bristle ends [7]. Toothbrushes with multilevel or angled bristles outperform conventional flat-trimmed bristles in plaque removal [8,9].

Nylon is the most widely used material for bristles, and studies have suggested a significant direct relationship between bristle diameter and stiffness in nylon toothbrushes [10]. However, the suboptimal water resistance of nylon bristles and their interaction with water over time leads to a decline in their tensile strength and modulus of elasticity [11].

Curaprox CS5460 is an ultra-soft toothbrush made of Curen® filaments with a hexagonal handle, an angled head, a cylindrical bristle shape, and round-ended bristles [12,13]. Each toothbrush head includes 5,460 individual bristles each with a diameter of 0.1 mm [13]. Along with the advantage of the design leading to optimal cleaning power at the correct angle, the material also exhibits a higher water resistance than nylon and maintains its stability even under wet conditions. Curen® filaments are made up of polyester filaments that absorb almost no water [14]. This characteristic allows for the utilization of finer filaments and a shorter head design while still ensuring their durability and effectiveness [15].

Given the lack of published evidence comparing nylon and Curen® filaments in toothbrushes, this in vitro study was conducted to compare the physicochemical properties of Curen® and nylon filaments.

## Materials and methods

Study specimens

**Figure 1:**
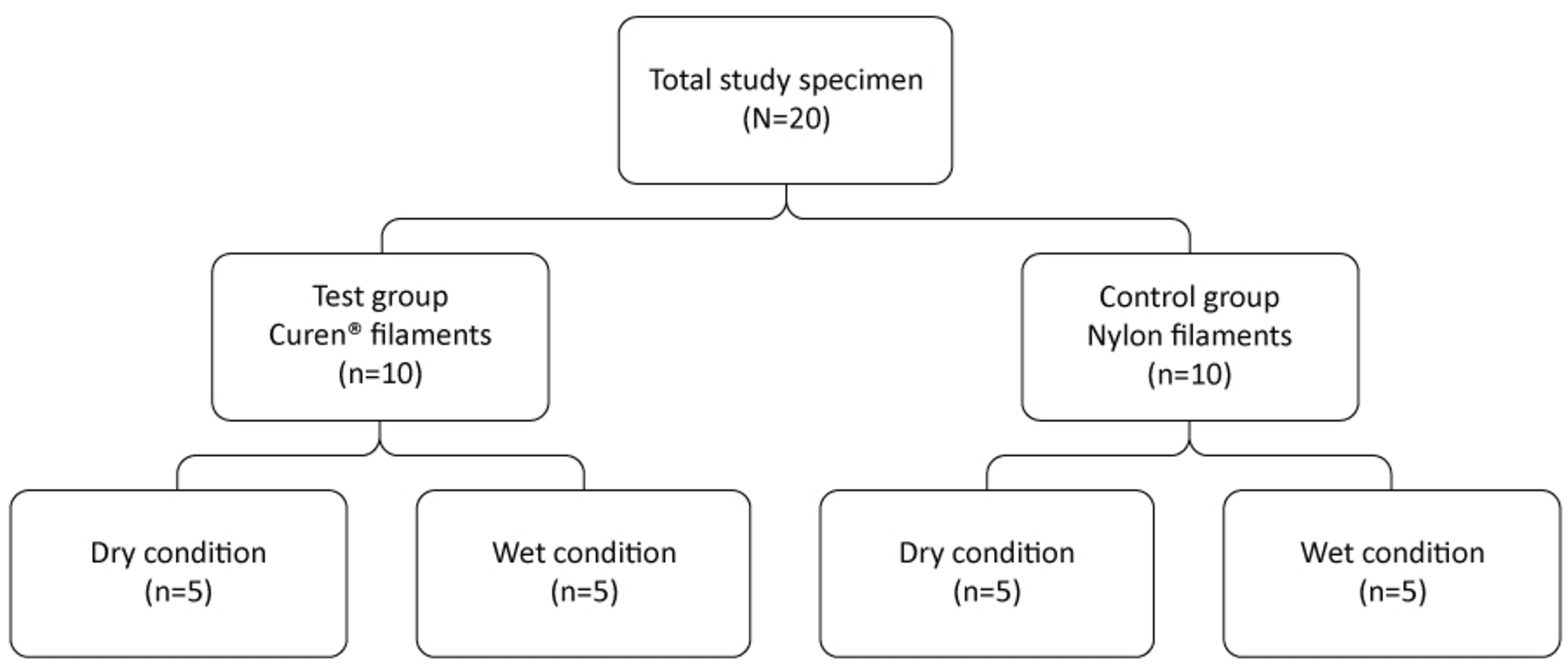
Distribution of specimens among the study groups

This in vitro study was approved by the Institutional Review Board (SRMU/M&HS/SRMDC/2023/002 dated February 15, 2023). The study was conducted on a total of 20 specimens, i.e., 10 specimens each of the Curaprox CS5460 toothbrush (premium Swiss oral care) with Curen® filaments as the test group and nylon filaments of a standard toothbrush as the control group. The diameters of the Curen® and nylon filaments selected were 0.1 mm to maintain uniformity. The distribution of the study specimen is shown in Figure 1.

Tensile strength of filaments and force-displacement analysis

Tensile strength testing was used to evaluate the modulus/stiffness of the filaments in both wet and dry conditions using a universal testing machine (UTM), ElectroPuls® E3000 (Instron®, Wycombe, United Kingdom). The force was evaluated by cutting one strand of a toothbrush bristle approximately 7 cm in length and positioning it using pneumatic tensile grips at the top and bottom ends of the UTM. The top end was drawn at intervals of 1 cm at a crosshead speed of 200 mm/minute to pull the specimen until failure occurred. The maximum force was recorded in newtons (N). Tensile stress was assessed using the force and cross-sectional area of the filament. Tensile strain represents the displacement or elongation of the filaments owing to the application of tensile force before fracture.

Force-displacement parameter was used as part of the tensile strength measurement. Force-displacement curves were plotted, and the maximum force and maximum displacement for each specimen were noted.

Brushing simulation

Brushing simulation was performed using the SD MECHATRONIK Toothbrush Simulator ZM-3.8 for 5,000 cycles (linear Y: 2500 cycles, circular: 2,500 cycles) and a load of 300 g, which mimics brushing for six months (Appendix A) [16]. The simulated brushing speed was 30 mm/second, and the stroke length was 8 mm. As per the protocol, the study was intended to simulate 3,000 brushing cycles, which represents 3.6 months of tooth brushing. However, to evaluate the outcome parameters beyond 3.6 months and understand the effects of the long-term use of Curen® and nylon filaments, the simulation was extended to 5,000 cycles. However, when assessed beyond 3,000 cycles, nylon filaments could not be visualized under the field emission scanning electron microscope (FE-SEM).

Abrasion potential of filaments

The abrasion potential of the filaments was evaluated before and after the brushing simulation (equivalent to six months of brushing). Six freshly extracted central incisors (n=3 each for the Curen® filament and nylon filament groups) were used to generate specimens. Teeth were inspected for imperfections on the surface. Teeth with cracks, caries, discolorations, or loss of hard tissue were excluded. Teeth were stored in 0.7% sodium chloride solution containing 0.1% thymol. Cylindrical specimens (6 mm in diameter and 2 mm high) were prepared using a trephine bur (Hager & Meisinger GmbH, Neuss, Germany). Only one specimen was prepared from each tooth. The tooth surfaces (crown region) were evaluated for surface roughness before and after the brushing simulation using a Nanosurf Nanite atomic force microscope (AFM; Aton Parr-Step 700; image size: 10 μm, resolution: 128 points/line, rotation: 45॰ using the cantilever Stat0.2LauD in contact mode).

Abrasion resistance of filaments

Two-inch long twisted-in-wire brushes (diameter: 112.903 mm) filled with the filament (diameter: 0.3048 mm) were rotated against the extracted natural tooth samples for 40 seconds under identical standard test conditions.

The specimens were then dried and stabilized on brass stubs using carbon tape and sputter-coated with platinum for 40 seconds and visualized using an FE-SEM (Jeol IT-800; magnification: 1,000x; resolution: 10 µm; scanning voltage: 10 kV; field of view: 366 × 274).

Bristle surface changes

Stereomicroscopy

Splaying of the filaments was assessed post-brushing by capturing images of the toothbrush bristles at a lower magnification of 10x on a Leica M205 C stereomicroscope, ensuring that the samples were positioned flat at their base. Modified evaluation criteria by van Nuss et al. were used for assessing the bristle surface changes by scoring the SEM images [17].

Micro-Computed Tomography Imaging (Volumetric Analysis)

Micro-computed tomography (micro-CT) imaging was performed using SkyScan 2214 (Bruker, Kontich, Belgium; scanning voltage: 85 kV; scanning current: 128 mA). Each sample was rotated by 360° during scanning. The micro-CT images were reconstructed using NRecon 2.1.0.2 and rendered into three-dimensional (3D) images of the toothbrush bristles using CTVox 3.3.0. The reconstructed micro-CT images of the samples produced detailed 3D renderings of the toothbrush bristles with precise measurements of the diameter and height of the individual bristles.

Nano CT Imaging

Nano CT imaging was performed using SkyScan 2214 (Bruker) software version 1.8 (source: Tungsten-Hamamatsu L10711 with two flat panels separated by 50-140 µm; source voltage: 85 kV; source current: 128 µA; exposure: 1,441 ms; resolution: 1,944 × 3,072, corresponding to a voxel resolution of 14 µm; applied filter: 1-mm copper resulting in 16-bit images; field-of-view camera pixel size: 14 µm). An X/Y ratio of 1.0010 was used to check the transaxial projections of the bristle surface changes in the 3D images.

Statistical analysis

Data were analyzed using SPSS software version 23 (IBMCorp., Armonk, NY, USA). Descriptive statistics were used to present the results of the tensile strength analysis. An independent sample t-test (two-tailed) was performed to assess the difference between the groups in the continuous variables of surface area and square mean roughness of the tooth surface. The homogeneity of variance was tested using Levene’s test.

## Results

Tensile strength testing

In both dry and wet conditions, Curen® filaments showed mean tensile strengths of 41.69 MPa and 35.18 MPa at forces of 1.38 N and 1.44 N, respectively, whereas nylon filaments exhibited significantly higher tensile strengths of 321.56 MPa and 325.44 MPa at forces of 9.66 N and 7.4 N, respectively (Table 1).

**Table 1:** Tensile strength and tensile strain of Curen® and nylon filaments groups **Statistically significant using unpaired t-test N, newtons; MPa, megapascals

		Curen® filaments group	Nylon filaments group	t	p-Value
Dry	Force (N)	1.38	9.66		
	Tensile strength (MPa)	41.69	321.56	-7.3	<0.001**
	Tensile strain (displacement) at break (standard) (%)	15.83	9.56	1.98	<0.001**
Wet	Force (N)	1.44	7.4		
	Tensile strength (MPa)	28.55	325.44	-5.59	<0.001**
	Tensile strain (displacement) at break (standard) (%)	18.61	22.76	1.14	<0.001**

For the nylon dry filaments, the maximum displacement was 2.6 mm with a maximum force of 0.01 kN, while for the Curen® dry filaments, it was 5.1 mm with a maximum force of 0.002 kN. In wet conditions, the nylon filaments showed a displacement of 7.2 mm with a maximum force of 0.005 kN, whereas the Curen® filaments exhibited a displacement of 5.1 mm with a force of 0.001 kN (Appendices B and C)

Abrasion potential

The percentage change in surface roughness in the Curen® filaments group for the crown region was -19.4%, while the change in the nylon filaments group was 92.3%. The Curen® filaments exhibited approximately 72.84% less abrasion compared to the nylon filaments. The roughness of the crown in the Curen® filaments group decreased by approximately 1.24 times (post-brushing vs. pre-brushing), while in the nylon filaments group, the crown roughness increased by 1.92 times. There was no significant difference between the Curen® and nylon filaments groups in the pre-brushing surface area of the crown region (46.45±11.76 vs. 45.55±36.28; p=0.967). Similarly, there was no significant difference between the two groups in the post-brushing surface area of the crown region (37.42±25.40 vs. 87.59±75.94; p=0.34) (Table 2).

**Table 2:** Mean roughness (surface area) of the crown region between the groups at pre-brushing and post-brushing SD, standard deviation; NS, not significant using unpaired t-test

Surface area	Groups	Number	Mean	SD	t	p-Value
Pre-brushing	Curen® filaments	3	46.45	11.76	0.04	0.97
	Nylon filaments	3	45.55	36.28		NS
Post-brushing	Curen® filaments	3	37.42	25.4	-1.09	0.34
	Nylon filaments	3	87.59	75.94		NS

Abrasion resistance of filaments

**Figure 2:**
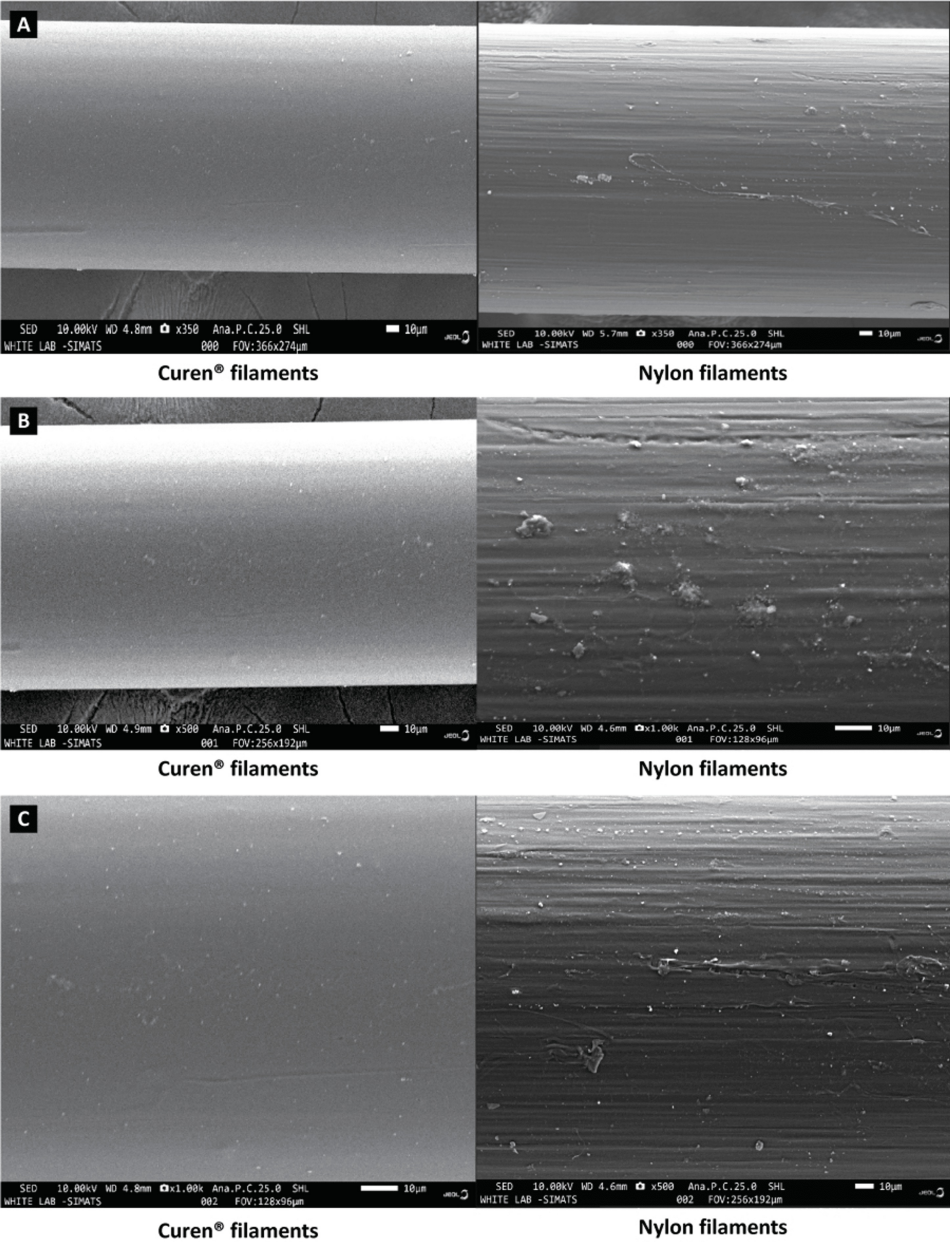
FE-SEM analysis. (A) Post-brushing 1,000 cycles. (B) Post-brushing 2,000 cycles. (C) Post-brushing 3,000 cycles. FE-SEM, field emission scanning electron microscopy

The FE-SEM analysis depicting microphotographs of the filaments after rotating against the extracted tooth surface obtained at 1,000x magnification showed an increase in scratches as the number of brushing cycles increased in both the Curen® filaments and nylon filaments groups (Figures 2A-2C). However, no difference was observed between the Curen® filaments and nylon filaments groups with respect to the abrasion resistance of the filaments. FE-SEM images were captured solely to visualize the microstructure of the filaments. Only the structural morphology was examined.

Bristle surface changes

Based on the modified evaluation criteria for scoring SEM images by van Nuss et al. [17] both nylon and Curen® filaments displayed similar surface changes. The scores as per van Nuss et al. [17] are presented in Table 3.

**Table 3:** Surface changes on nylon and Curen® filaments Interpretation of score Score 1: New appearance, smooth bristle surface, bristles standing straight and even, no split ends. Score 2: Slight signs of wear, bristles standing straight and even, irregular bristle ends, little surface roughness, no split ends.

Group	Sample	Post-brushing (1,000 cycles)	Post-brushing (2,000 cycles)	Post-brushing (3,000 cycles)	Post-brushing (5,000 cycles)
Curen^®^ filaments	1	2	2	2	2
	2	2	2	2	2
	3	2	2	2	2
	4	2	2	2	2
	5	2	2	2	2
Nylon filaments	1	2	2	2	2
	2	2	2	2	2
	3	2	2	2	2
	4	2	2	2	2
	5	2	2	2	2

Stereomicroscopy

**Figure 3:**
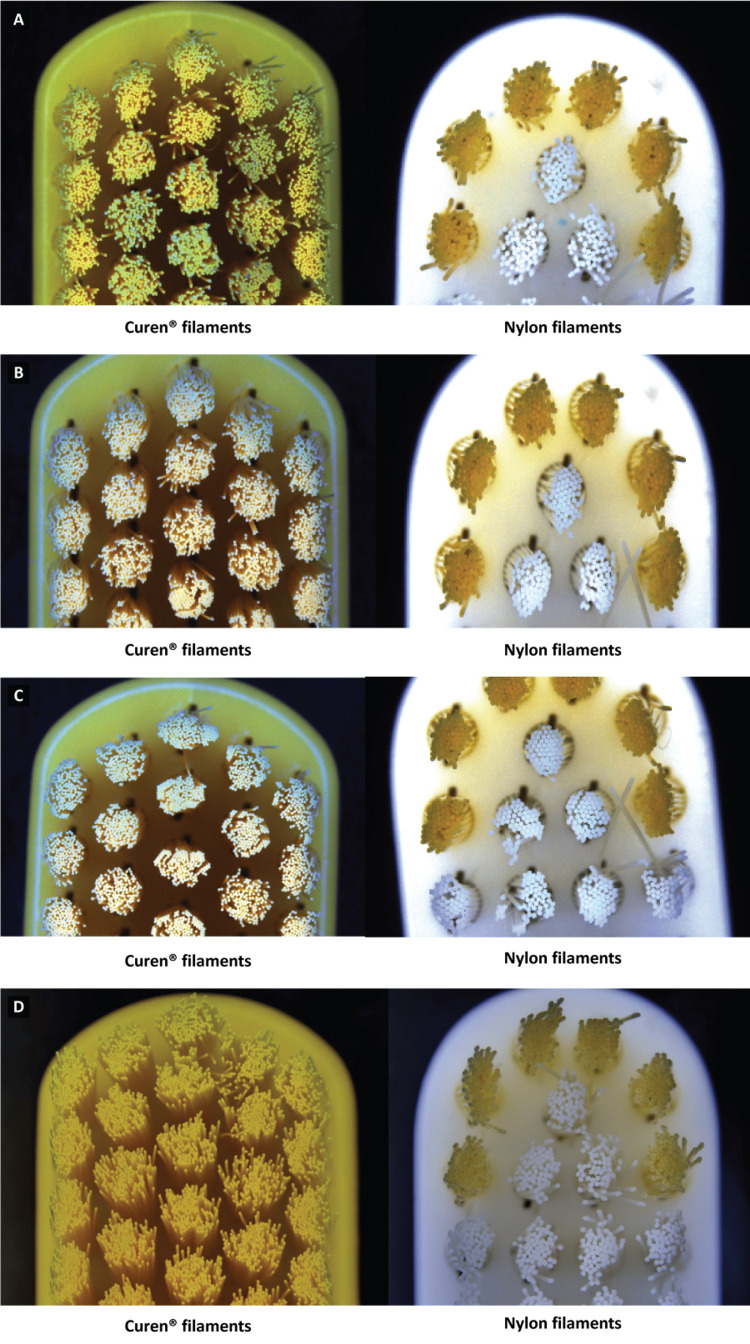
Stereomicroscopic images of toothbrush filaments post-brushing. (A) Post-brushing 1,000 cycles. (B) Post-brushing 2,000 cycles. (C) Post-brushing 3,000 cycles. (D) Post-brushing 5,000 cycles.

The stereomicroscopic images indicate that both nylon and Curen® filaments display similar splaying after each stage of the brushing simulation. However, when they were subjected to 3,000 and 5,000 cycles of simulated brushing, the Curen® filaments displayed 30% less splaying compared to the nylon filaments (Figures 3A-3D).

Micro-CT Imaging of Curen® Bristles

The pre- and post-brushing base diameters of the Curen® bristles were 1.784 mm and 2.035 mm, respectively, with a difference of 0.251 mm and percentage change of 14.1%, and top diameters were 2.157 and 2.390 mm, respectively, with a variation of 0.233 mm and percentage of 10.8% (Table 4). The pre- and post-brushing heights of the Curen® bristles were 12.0 mm and 11.95 mm, respectively with a difference of -0.05 mm and a percentage change of -0.4%. The pre- and post-brushing bristle angulation changed from 90° to 88.10°, with a percentage change of -2.1%. Micro-CT scan was performed only for Curen® filaments as the nylon filaments were not visible after reconstruction even after scanning with low power setup.

**Table 4:** Volumetric analysis of toothbrush bristles

Groups	Pre-brushing (A)	Post-brushing (5,000 cycles) (B)	Difference (B – A)
Height (mm)	12.0	11.95	–0.05
Top diameter of bristles (mm)	2.157	2.390	0.233
Base diameter of bristles (mm)	1.784	2.035	0.251

**Figure 4:**
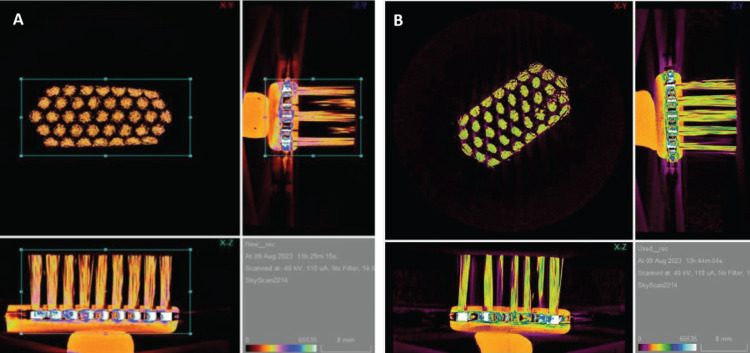
Reconstructed micro-CT imaging of Curen® bristles. (A) Pre-brushing. (B) Post-brushing 5,000 cycles.

For the image reconstruction, 360° rotation was initialized with a rotation step of 1.000°. The average pre-brushing and post-brushing scanning times were 11h:52m:58s and 13h:14m:05s, respectively (Figures 4A, 4B).

**Figure 5:**
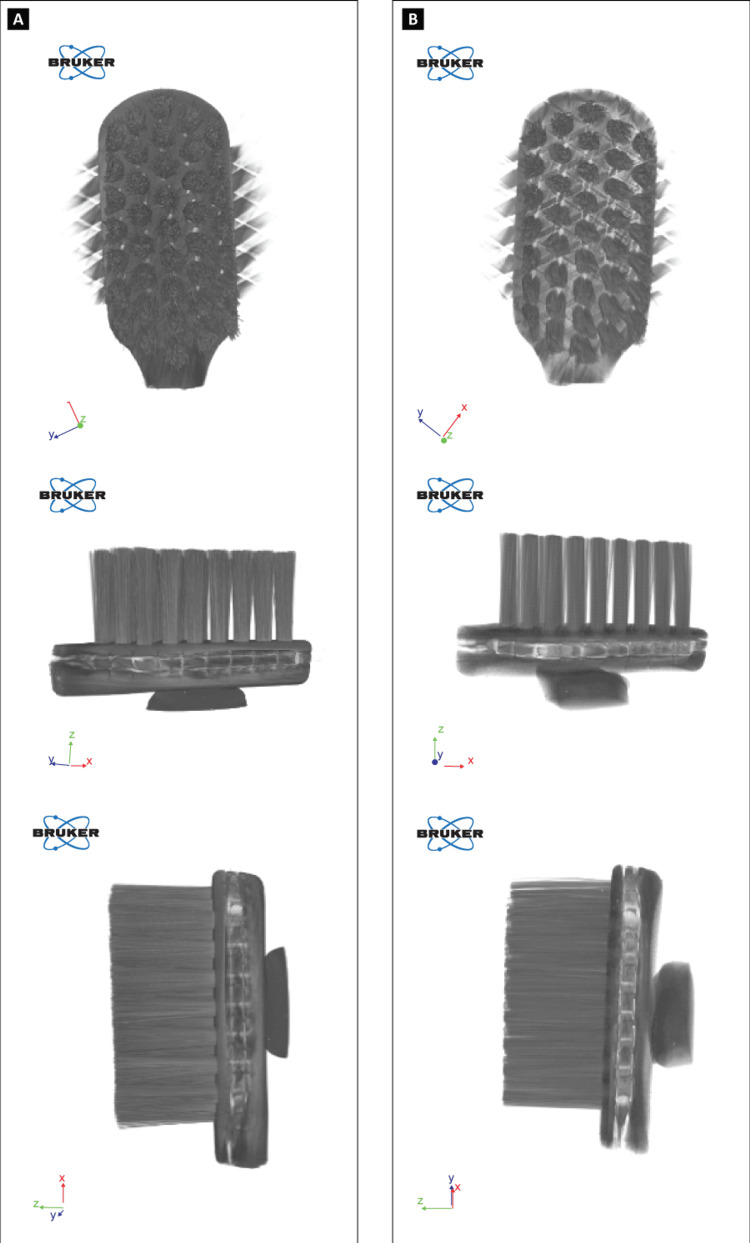
Nano-CT images of the Curen® bristles. (A) Pre-brushing. (B) Post-brushing 5,000 cycles.

The nano-CT images of the Curen® bristles are shown in Figures 5A, 5B.

## Discussion

This in vitro investigation comparing the physicochemical properties of Curen® and nylon filaments in toothbrushes showed that Curen® filaments had a lower tensile strength than nylon filaments. Abrasion potential and resistance to abrasion did not differ significantly between Curen® and nylon filaments. Microscopy analyses of bristle surface changes did not reveal major changes after brushing simulation for 5,000 cycles. Further, the splaying observed was 30% less with Curen® compared to nylon filaments.

Toothbrushing is reported as the most common cause of abrasion on the cervical edges of teeth [18]. Besides toothbrushing technique, brushing force and frequency, duration of brushing, and brush type, the stiffness of filaments used in the toothbrush is a major contributor to abrasion [18]. The greater the stiffness, the greater the risk of toothbrush abrasion. The present study compared the stiffness of Curen® filaments with conventional nylon filaments (high tensile strength of 250 MPa according to Matayoshi et al. [19] and Chandramohan et al. [20]). The Curen® filaments demonstrated lower tensile strength than nylon filaments, which aligns with the findings from a study by Fattal et al. [13], in which bristles with Curen® filaments outperformed those made of nylon filaments in terms of bristle quality after six months of use, which was determined based on the number of unsatisfactory bristles, i.e., sharp and uneven edges with a rough surface [13]. The lower tensile strength of Curen® filaments likely causes their reduced displacement and forces compared to nylon filaments [6]. Greater brushing forces along with faulty brushing methods can lead to gingival recession and cervical abrasion of tooth surfaces [6,21]. The low tensile strength of Curen® filaments over nylon filaments is less likely to cause damage to the enamel or gingiva, thereby providing a gentle cleaning experience [22] and highlighting the superiority of Curen® filaments over nylon filaments.

The direction of the brushing, the number of filaments in the tuft, and the spacing of the tufts affect the entry and exit of particles [21]. Harder toothbrush bristles retain abrasive particles for a longer duration, while wet toothbrushes accelerate bristle wear and reduce firmness, decreasing effectiveness [23]. In the present study, toothbrushes with nylon bristles were stiffer in both wet and dry states than toothbrushes with Curen® bristles, thereby indicating superior effectiveness of Curen® bristles over nylon bristles.

Bristle softness is inversely proportional to the abrasive potential of a toothbrush [24]. To build on the results of tensile strength testing, the abrasion potential of the two filaments was studied using a toothbrush simulator wherein filaments were subjected to linear (2,500 cycles) and circular (2,500 cycles) motion for a total of 5,000 cycles with a standardized brushing force of 300 g, mimicking six months of brushing [25]. Atomic force microscopic analyses revealed no significant differences in tooth surface roughness before and after simulated brushing for 5,000 cycles in both the Curen® and nylon filaments groups. Though not significant, the enamel roughness caused by Curen® filaments (decreased by 1.24 times) was lower than that caused by nylon filaments (increased by 1.92 times). This may be attributed to the flexibility of Curen® filaments, allowing them to conform to the contours of the teeth and gingiva without exerting excessive force, thereby minimizing the risk of enamel abrasion. Thus, over time, toothbrushes with ultrasoft filaments, such as Curen®, may be less abrasive, and cause less enamel abrasion and gingival recession than toothbrushes with nylon filaments [26]. Additionally, Curen® filaments, being less abrasive, could benefit individuals experiencing enamel sensitivity, making them suitable for special cases.

Changes in bristle surface can hinder plaque removal by limiting access to difficult-to-reach surfaces, while worn sharp ends of bristle can cause gingival abrasion and inflammation [27]. In the present study, there were minimal changes in the diameters from the base to the tip, height, and bristle angulation of the Curen® filaments after simulated brushing. Bristle diameter largely determines bristle stiffness [10]. Manual toothbrushes feature flat handles and densely packed, uniform-length bristles [28]. Changes in bristle height compromise cleaning efficacy. Nylon filaments, with their larger diameter, good stiffness, and abrasiveness, effectively remove plaque by increasing tooth surface contact. However, they simultaneously result in more abrasion and dentin loss [20,21,26]. As the Curen® filaments have less tensile strength than nylon filaments, they tend to have greater flexibility during brushing, and this may help in avoiding permanent deformations.

Bristle splaying refers to a condition when bristles curve and diverge from their original alignment, reducing efficacy in plaque removal [29,30]. Higher filament flexibility reduces bristle splaying and enables access to hard-to-reach areas, crucial for maintaining adequate oral hygiene [31]. An in vitro study assessing the plaque removal efficacy of manual toothbrushes with different design parameters reported that toothbrushes with ultra-soft/soft bristles featuring a smaller filament diameter removed plaque efficiently across all surfaces of the tooth [4]. The nano- and micro-CT images of the present study demonstrated 30% less splaying of the Curen® filaments compared to nylon filaments after simulated brushing for 5,000 cycles. As Curen® filaments show higher abrasive resistance and less splaying than nylon filaments, toothbrushes with Curen® filaments may be more durable than those with nylon filaments. The volumetric analysis further substantiates this by demonstrating minimal changes in Curen® filaments after 5,000 cycles of brushing. These results indicate that a toothbrush with Curen® filaments, even after 5,000 cycles of brushing, is comparable in condition to a non-used toothbrush. The American Dental Association recommends replacing toothbrushes every three to four months or sooner if the bristles show splaying, as splaying reduces the effectiveness of toothbrushes [8]. However, based on the study by Di Fiore et al. [16], demonstrating 100,000 brushing strokes simulating 10 years of clinical wear, it can be hypothesized that Curen® filament toothbrushes exhibited minimal splaying even after 5,000 cycles, simulating six months of brushing, and thus can be used beyond three months with acceptable effectiveness when compared to nylon filament toothbrushes. Consequently, toothbrushes with Curen® filaments could be recommended by dentists as a longer-lasting choice for oral hygiene.

The limitation of this study includes the in vitro nature of the research, which may not accurately reflect in vivo conditions. For example, biofilm formation, which cannot be studied in vitro, has the potential to alter the immunological system of the individual and result in dental plaque formation, plaque-induced gingivitis, and related diseases. Clinical studies need to be conducted to correlate the findings of this in vitro study with clinical findings. Thus, additional real-world studies on the efficacy of Curen® filaments could contribute to toothbrush recommendations for plaque reduction and enamel mechanical wear apart from few available studies. In addition, real-world studies among different age groups with altered enamel morphology (e.g., non-carious lesions) would provide more data, enabling dentists to make decisions based on scientific evidence.

## Conclusions

The present study has effectively analyzed the distinct properties of Curen® and nylon filaments that can inform personalized toothbrush recommendations. While nylon filaments offer high tensile strength, Curen® filaments showed lower tensile strength, minimal splaying and abrasion potential, and good filament abrasion resistance after simulating 1,000-5,000 brushing cycles. The lower tensile strength and abrasion potential of Curen® filaments offer flexibility and adequate cleaning of hard-to-reach areas with minimal force, thereby preventing enamel abrasion and gingival recession. The good abrasion resistance of Curen® filaments contributes to the longevity of the toothbrush up to six months. However, further clinical trials and real-world studies are crucial for translating these in vitro findings into practical toothbrush recommendations, especially in addressing plaque reduction and minimizing enamel abrasion and gingival recession. The outcomes of future research will further benefit dental professionals and guide individuals in choosing toothbrushes tailored to their specific needs.

## Figures and Tables

**Figure 6:**
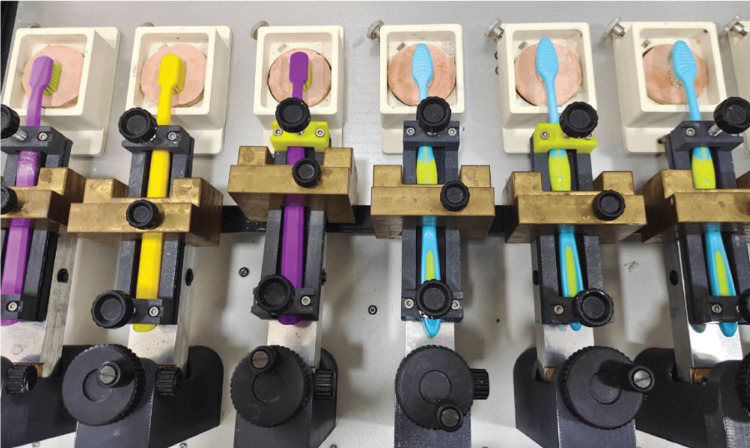
Apparatus used for brushing simulation (SD MECHATRONIK Toothbrush Simulator ZM-3.8)

**Figure 7:**
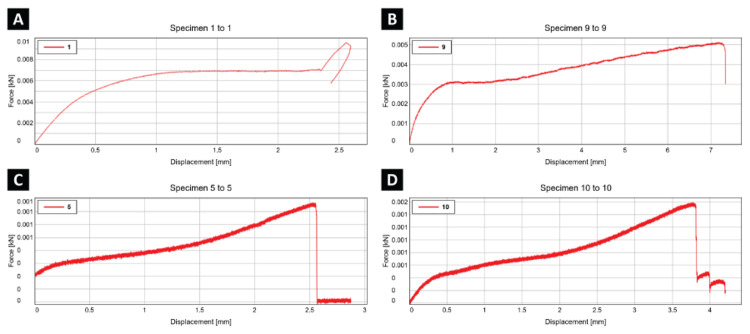
Representative force–displacement curves. (A) Nylon filaments in the dry condition. (B) Nylon filaments in the wet condition. (C) Curen® filaments in the dry condition. (D) Curen® filaments in the wet condition.

## Appendices

Appendix A 

Appendix B

Appendix C 

**Table 5:** Force–displacement data for each specimen

Specimen no.	Specimen name	Max force (kN)	Max displacement (mm)
1	Nylon Dry	0.01	2.6
2	Nylon Dry	0.008	2.3
3	Nylon Dry	0.01	2.5
4	Nylon Dry	0.008	2.3
5	Nylon Dry	0.01	2.5
6	Curen^®^ Dry	0.001	3.5
7	Curen^®^ Dry	0.001	2.6
8	Curen^®^ Dry	0.002	5.1
9	Curen^®^ Dry	0.001	2.6
10	Curen^®^ Dry	0.002	5.0
11	Nylon Wet	0.008	5.2
12	Nylon Wet	0.008	4.4
13	Nylon Wet	0.008	5.2
14	Nylon Wet	0.008	4.4
15	Nylon Wet	0.005	7.2
16	Curen^®^ Wet	0.002	3.7
17	Curen^®^ Wet	0.001	5.1
18	Curen^®^ Wet	0.002	4.3
19	Curen^®^ Wet	0.002	3.7
20	Curen^®^ Wet	0.002	4.3
